# Effects of Erythrocytapheresis Procedures on Delayed Bone Marrow Conversion in Sickle Cell Disease

**DOI:** 10.1007/s44228-022-00022-6

**Published:** 2022-11-16

**Authors:** Emily Popham, Karen Moeller, Ashok Raj

**Affiliations:** 1 University of Louisville School of Medicine, Louisville, KY USA; 2 Norton Children’s Hospital, Louisville, KY USA; 3 Division of Pediatric Hematology/Oncology, Norton Children’s Hospital, University of Louisville, Louisville, KY USA

**Keywords:** Sickle cell disease, Erythrocytapheresis, Neuroimaging, Skeletal abnormalities

## Abstract

The imaging appearances of the skeletal system have been well documented in sickle cell disease (SCD) but there is limited information about the impact of SCD treatments on skeletal abnormalities. We present two patients with SCD maintained on long-term erythrocytapheresis and the changes to their skeletal abnormalities on neuroimaging with this treatment. We observed a reversal of the bone marrow conversion process and the skull appearance was age appropriate without any radiographic findings of iron overload in the patients.

## Introduction

Sickle cell disease (SCD) is the most common structural hemoglobinopathy and is caused by a mutation that causes the characteristic sickling of erythrocytes that leads to several short- and long-term complications [1–3]. The frequency and severity of these complications vary based on the type of SCD, but include hemolytic anemia, vaso-occlusive crises, acute chest syndrome, splenic sequestration, osteomyelitis, stroke, and chronic organ damage [1, 2]. The chronic anemia caused by SCD can also lead to bone marrow hyperplasia and persistence of hematopoietically active red bone marrow [4]. There are many disease-modifying therapies for SCD, including erythrocytapheresis, an automated red cell exchange procedure that removes red cells containing hemoglobin S (HbS) from the patient while simultaneously replacing that same volume with red cells free of HbS [5]. Long-term erythrocytapheresis (LTE) is used to treat complications of SCD and is rarely used in non-sickle cell disorders [5]. Indications for erythrocytapheresis in SCD include primary and secondary prevention of stroke, recurrent acute chest syndrome, and multiorgan failure [5]. LTE is associated with a reduced risk of iron overload that is associated with long-term simple red cell transfusions [5]. Red cell exchange does this by diluting HbS without raising hematocrit, which keeps iron stores steady and prevents iron deposition in organs such as the liver, head and neck bone marrow, and salivary glands [4, 5].

## Case Presentation

Patient A is a 15-year-old African American male with sickle cell anemia (Hb SS disease) who has made multiple trips to the emergency room for acute chest syndrome and pain crises. He has a history of pancreatitis with cholelithiasis and has had his gallbladder removed. He began taking coumadin after being diagnosed with chronic thrombosis of the brachiocephalic vein extending into the superior vena cava. He has been maintained on LTE since 8 years of age due to increased transcranial Doppler velocity and magnetic resonance imaging (MRI) findings suggestive of central nervous system (CNS) vasculopathy. He has a history of transfusion reactions and requires washed red blood cells and diphenhydramine (Benadryl) treatment before transfusions.

**Fig. 1:**
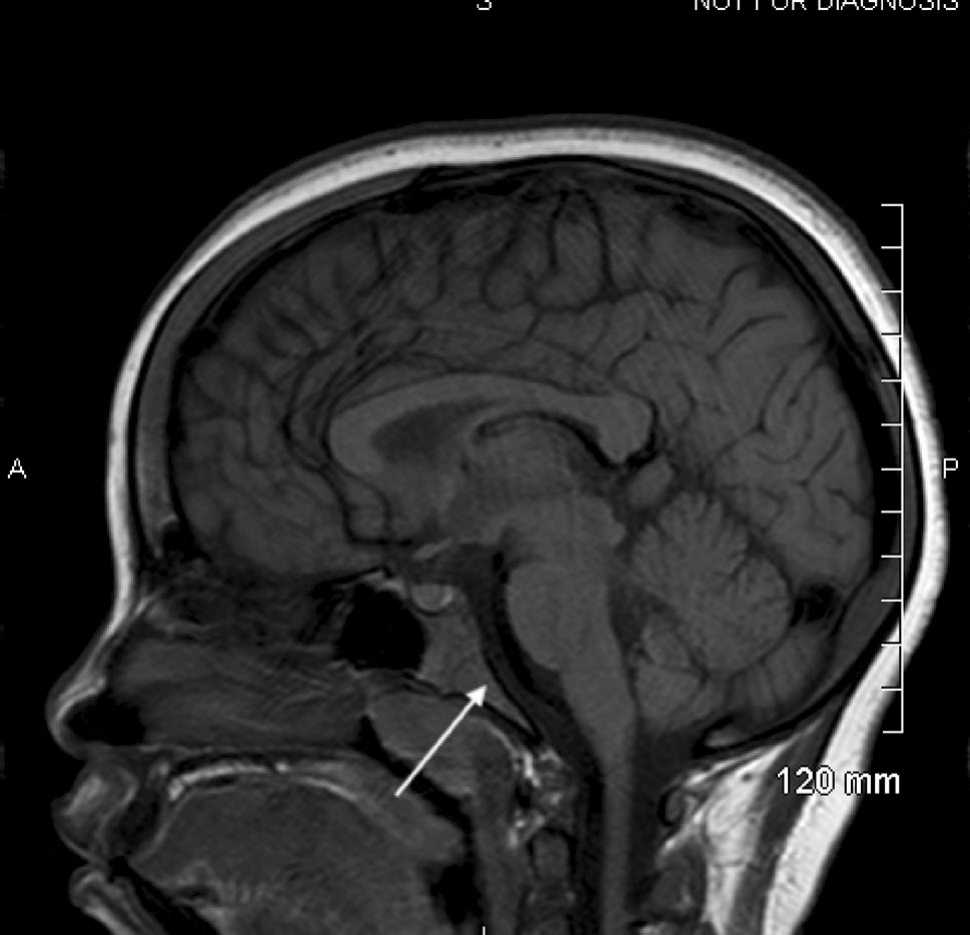
The calvarium and clivus have a low T1 signal

**Fig. 2:**
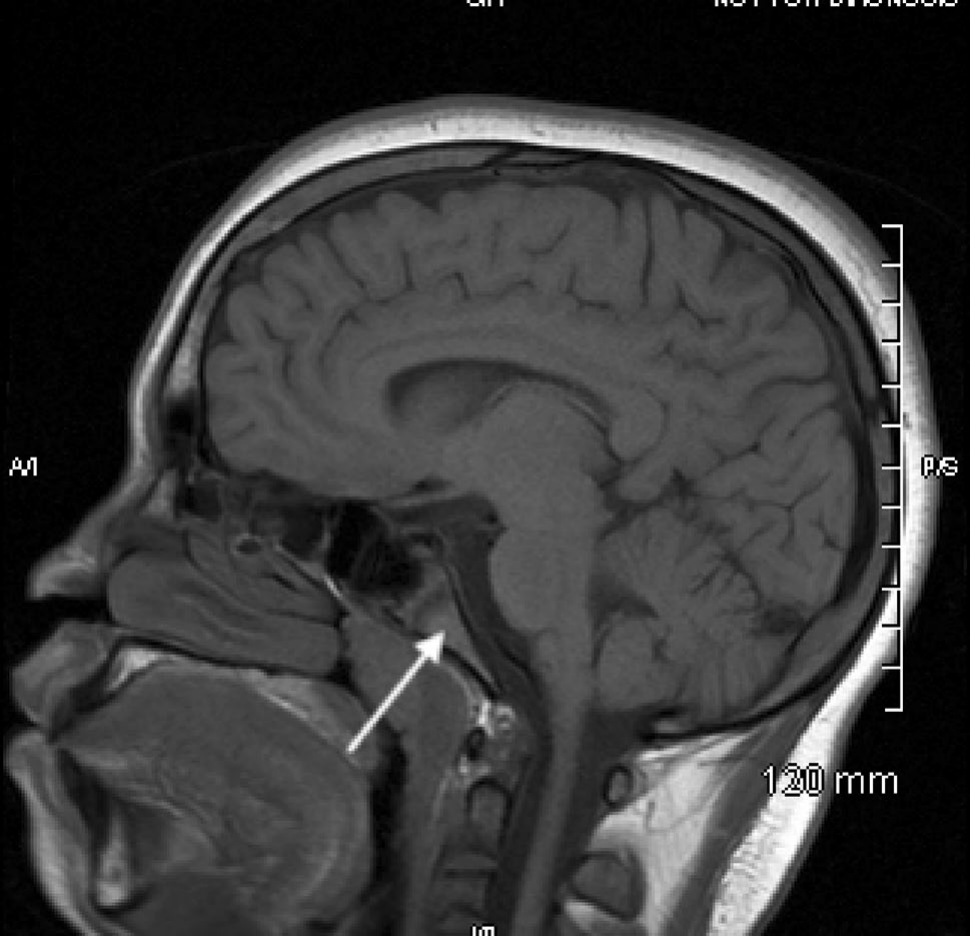
Both the calvarium and clivus have increased T1 signal

As part of the assessment of CNS vasculopathy, he has had routine MRIs for several years. Previous findings showed narrowing of the left supraclinoid internal carotid artery as well as a decreased bone marrow signal in the T1- and T2-weighted images that was consistent with SCD osseous changes. A follow-up MRI showed normal appearance of the bone marrow and no narrowing of the left supraclinoid internal carotid artery that had been noted on previous scans. A Doppler ultrasound performed the same year showed no evidence of the previously seen thrombosis of the brachiocephalic vein and no flow obstruction. His baseline sagittal T1 MRI and one taken 4 years later can be seen in Figs. 1 and 2.

**Fig. 3:**
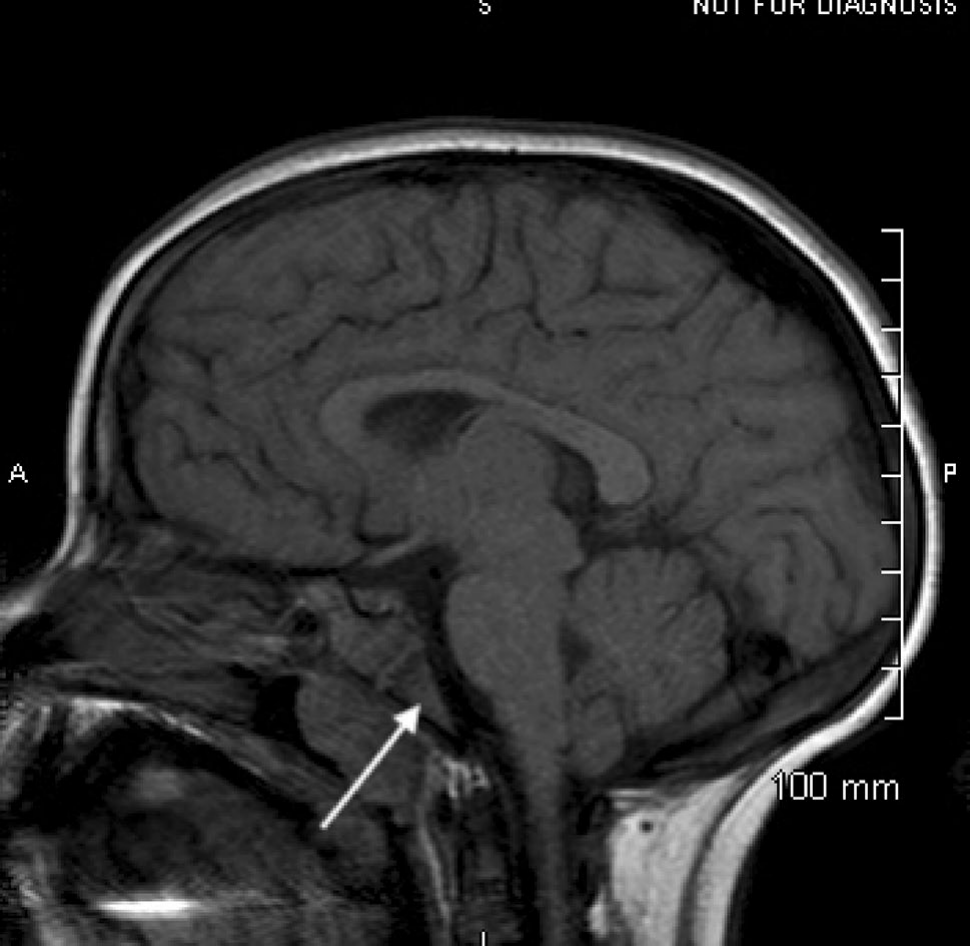
The visualized osseous structures have a dark T1 signal

**Fig. 4:**
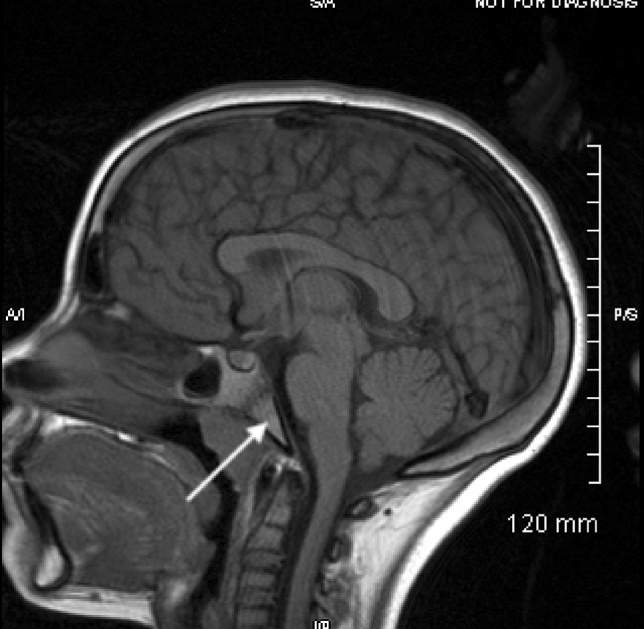
The visualized osseous structures have a high T1 signal

Patient B is a 12-year-old African American female with sickle beta 0 thalassemia (Hb Sβ^0^ disease) with a history of splenic sequestration that led to a splenectomy at age 5. She has been maintained on LTE since 6 years of age secondary to an increased transcranial Doppler velocity and a narrowing of the left middle cerebral artery seen on MRI. Clinical course on erythrocytapheresis has been uncomplicated. Erythrocytapheresis was started after treatment with hydroxyurea was discontinued due to non-adherence. She started coumadin in December 2017 due to narrowing and non-obstructive thrombosis of the right subclavian vein; she is also taking Adderall and guanfacine for attention-deficit/hyperactivity disorder. As part of an assessment for CNS vasculopathy, routine MRIs have been obtained throughout treatment; the baseline brain MRI showed increased signal in the osseous structures on T1- and T2-weighted images that were later resolved in an MRI that showed a normal signal. Her sagittal baseline T1 MRI and one taken 4 years later can be seen in Figs. 3 and 4.

## Discussion

Bone marrow is typically classified by its gross colorization into red or yellow marrow. Red bone marrow, also known as hematopoietic marrow, gets its color from the hemoglobin present in red blood cells and their precursors [6, 7]. Hematopoietic marrow actively creates new blood cells and is made up of about 40% water, 40% fat, and 20% protein [6, 7]. Yellow bone marrow, also known as fatty marrow, is composed of about 15% water, 80% fat, and 5% protein and is much less involved in hematopoiesis [6, 7]. Due to their differing compositions, hematopoietic and fatty marrow have different intensities when viewed using MRI [6, 7]. The higher concentration of adipocytes in fatty marrow gives it a higher signal intensity than that of hematopoietic marrow; the signal intensity of fatty marrow is typically comparable to that of subcutaneous fat, whereas the intensity of hematopoietic marrow is similar to that of nearby muscle [7].

Throughout a person’s life, the proportion of hematopoietic and fatty marrow varies, with the most rapid change occurring in the first 2 decades of life; the normal progression of hematopoietic marrow to fatty marrow has been outlined using their varying signal intensities on MRI [7]. The majority of bone marrow in infants is the hematopoietic variety, and the process of bone marrow conversion begins shortly after birth [6, 7]. In the appendicular skeleton, marrow conversion typically starts distally and progresses centrally [6, 7]. In the axial skeleton, marrow conversion is on average more gradual with each region following a distinct pattern [6, 7]. The most rapid transition of hematopoietic to fatty marrow in the skull typically occurs between birth and 5 years; conversion occurs in the skull base and facial bones before the calvarium [7]. Fatty marrow is first seen in the anterior sphenoid bone and marrow conversion in the skull base is typically finished by age 7 [7]. Hematopoietic marrow replacement in the facial bones begins in the mandible and maxilla before progressing to the hard palate, paranasal sinuses, and calvarium [7]. Changes in signal intensity are first seen in the frontal region of the calvarium at about 1 year of age and spread to surrounding regions in the next few years; a low signal intensity is abnormal after 7 years and the transition is typically finished by 15 years of age [7]. In the spine, signal intensity begins to increase between 6 months and 1 year and is typically hyperintense by age 5 [7]. The normal adult pattern is typically reached by about 25 years of age, characterized by the presence of hematopoietic marrow in the vertebral bodies, sacral bone, medial parts of hip bones, and articular ends of humeral and femoral bones [6–8].

In cases of increased oxygen demand or decreased oxygen delivery, bone marrow reconversion is possible; this can be seen in heavy smokers, athletes with a high oxygen demand, those with obesity, those with heart failure, diabetics, anemic patients, and those treated with hematopoietic growth factors [7–9]. When bone marrow reconversion occurs, it begins in the axial skeleton and moves through the appendicular skeleton proximally to distally, reversing the pattern seen in bone marrow conversion [6–8]. When viewing physiologic bone marrow reconversion on MRI, it will typically be symmetrical, will spare the articular ends, and will follow the characteristic pattern of beginning in the axial skeleton, which can help to differentiate it from pathologic conditions [8]. If the hematopoietic demand is high enough, red marrow hyperplasia or extramedullary hematopoiesis may occur, which can lead to additional complications [6]. An analysis of craniofacial bone marrow in patients with SCD found MRI changes consistent with increased hematopoietic marrow and decreased fatty marrow compared with control patients, as well as an increase in craniofacial bone marrow volume, suggesting delayed red-to-yellow marrow conversion [9]. These changes were hypothesized to be caused by increased erythropoiesis due to chronic hemolytic anemia [9]. The study also suggested that patients who have received repeated red cell blood transfusions could show changes in T2 times due to iron deposition [9].

As can be seen in the above images, there is a decreased signal in the skull base, calvarium, and visualized upper cervical vertebrae in the baseline images compared with the images taken 4 years later in both patient A and patient B. The hypointense signals and symmetric changes that follow the expected reconversion pattern in the baseline MRIs are indicative of persistent hematopoietic bone marrow caused by the chronic anemia that is associated with SCD. However, as seen in later MRIs, there was an increase in signal intensity associated with fatty bone marrow. While these changes are expected as children age, the earlier MRIs are representative of an already delayed bone marrow conversion process. Through LTE, these changes were reversed and now appear age appropriate.

The clinical significance of these findings could have relevance considering the studies suggesting an increased risk of malignant neoplasms such as myeloid leukemia in SCD [10, 11]. Although the cause of this association is speculative, the hypotheses considered include the effects of constant hematopoietic hyperplasia, stimulated by a hemolysis induced cytokine release at a much higher rate than normal, and a role for cytokine signaling in early myeloid leukemia pathogenesis. The limited information on SCD and cancer does not include patients maintained on LTE. Our findings could lead to a study of bone marrow progenitors in patients with SCD who are maintained on LTE.

## Abbreviations

SCD: Sickle cell disease
HbS: Hemoglobin S
LTE: Long-term erythrocytapheresis
MRI: Magnetic resonance imaging
T1: Longitudinal relaxation time
T2: Transverse relaxation time
CNS: Central nervous system

## Declarations

### Availability of Data and Materials

Data and materials are available for review.
